# Myomodulation with Injectable Fillers: An Update

**DOI:** 10.1007/s00266-020-01768-1

**Published:** 2020-08-05

**Authors:** Maurício de Maio

**Affiliations:** MD Codes™ Institute, Rua Santa Justina, 660 - cjs 121 e 124, Vila Olímpia, São Paulo, SP 04545-042 Brazil

*Level of Evidence V* This journal requires that authors assign a level of evidence to each article. For a full description of these Evidence-Based Medicine ratings, please refer to the Table of Contents or the online Instructions to Authors www.springer.com/00266.

“Myomodulation with Injectable Fillers: An Innovative Approach to Addressing Facial Muscle Movement” [1] was presented as a theoretical discussion of the concept of using injectable hyaluronic acid (HA) fillers to modulate the action of mimetic muscles to improve facial appearance. Traditionally, excessive muscle contraction has been addressed using neurotoxins (chemical myomodulation), with HA fillers used in conjunction to add volume and fill folds and wrinkles [2, 3]. However, extensive experience using HA fillers to treat patients with facial palsy, structural deficits, and bone and/or soft tissue loss associated with aging has shown that filler treatment can alter interactions between facial structure and muscle movement and balance action within muscle groups to modify appearance on both animation and at rest. Chemical myomodulation is used to reduce muscle contraction, whereas HA fillers can act via mechanical myomodulation to either increase or reduce muscle action (Fig. 1a).

**Fig. 1:**
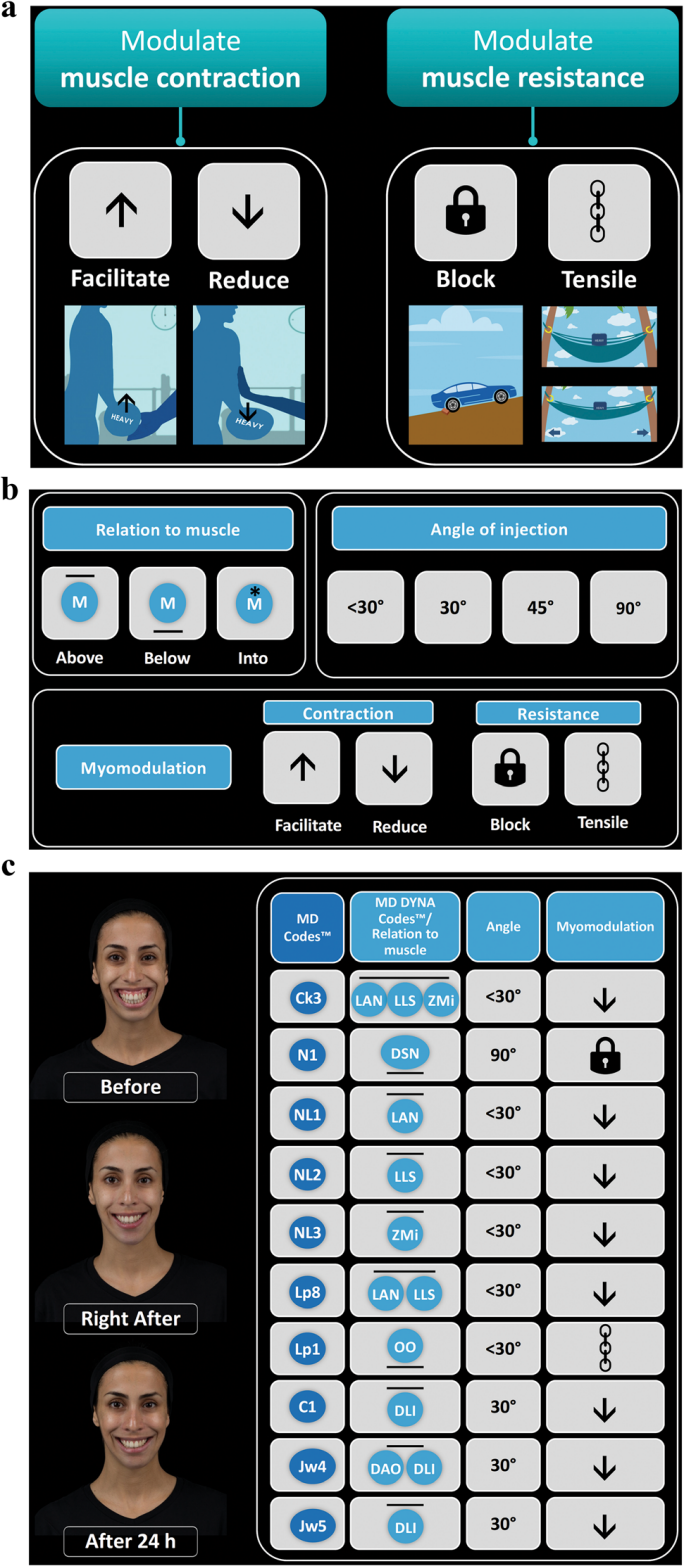
MD Codes and MD DYNA Codes for implementing myomodulation in clinical practice. **a** Mechanical myomodulation with HA fillers. **b** The language of the MD DYNA Codes. **c** HA filler treatment using the MD Codes and MD DYNA Codes. Case: 21-year-old woman with gummy smile. *C* chin, *Ck* cheek, *DAO* depressor anguli oris, *DLI* depressor labii inferioris, *DSN* depressor septi nasi, *HA* hyaluronic acid, *Jw* jowl, *LAN* levator alaeque nasi, *LLS* levator labii superioris, *Lp* lip, *M* muscle, *N* nose, *NL* nasolabial, *OO* orbicularis oris, *ZMi* zygomaticus minor

As discussed in the original paper, filler treatment can be used to correct structural deficiency, facilitate muscle action, and create an obstacle to extreme muscle excursion and depressor contraction. Where contraction is weakened due to congenital structural deficiency or volume loss with aging, mechanical myomodulation can facilitate action by providing support under the muscle between its origin or insertion (creating a fulcrum or pulley effect), or improve tensile strength by increasing distance between the origin and insertion, thereby reducing slack. Where muscle action is excessive, it can be reduced by adding tissue resistance above the muscle or injecting directly into or beneath the muscle near its origin or insertion to create a mechanical block. As a theoretical paper, the discussion of these concepts was limited by a lack of experimental evidence for proposed mechanisms underlying observed effects, and the effectiveness of treatment based on myomodulation has not yet been assessed in clinical studies [4]. However, although the posited mechanisms remain to be tested, the ideas presented in the paper can be of value to clinicians practicing facial aesthetics and rehabilitation, challenging them to reconsider how they plan and carry out HA filler and neurotoxin treatments.

The current focus of my work has grown directly out of this understanding of the value of myomodulation with HA fillers. In forthcoming publications, clinical tools developed for employing mechanical myomodulation in practice will be described. The MD DYNA Codes™ and their associated MD Codes™ were conceived as a symbolic language to refine and systematize facial assessment and injection technique. The MD Codes constitute a set of precisely defined placement points and technical details that can serve as injection guidelines, to be tailored for each individual patient. They divide the face into structural units and, through the use of symbols, depict target structures, injection technique, product choice, and danger areas for each unit. The MD Codes can be used in the planning and sequencing of treatment steps to best address a patient’s unfavorable attributes (a saggy appearance or a tired, sad, or angry look) and enhance positive attributes (an attractive, younger, more contoured, and feminine or masculine appearance), and to communicate best practices in aesthetic treatments. The MD DYNA Codes detail targeted muscles, and the desired effects of HA filler injection suggest specific HA placement for achieving those effects (above, below, or into the muscle), and provide required technical details (e.g., angle of needle or cannula) for making the injection (Fig. 1b). That is, the MD DYNA Codes are simply a technical strategy for implementing myomodulation. They are used with the MD Codes such that MD Codes define the facial structure to be addressed, while the MD DYNA Codes describe the target muscles and the planned change in function.

The MD Codes and MD DYNA Codes in no way replace the skill and judgment of an experienced practitioner. Knowledge of facial muscle anatomy is essential, and insightful understanding of functional groups and muscle synergism/antagonism is necessary for the effective use of HA filler placement for mechanical myomodulation. But the expectation is that these tools may reduce variability in patient outcomes and provide clinicians with a new treatment approach. For the future, a more detailed article outlining MD Codes and MD DYNA Codes for myomodulation is planned, as is a more formal injection guide. Here, a brief case is presented to illustrate the principles of their use.

A 21-year-old Caucasian woman presented with gummy smile in both the upper and lower dental arcade (Fig. 1c, top left) resulting from excessive movement of the upper lip levators (levator alaeque nasi [LAN], levator labii superioris [LLS], and zygomaticus minor [ZMi]), depressor septi nasi (DSN), and the lower lip depressors (depressor labii inferioris [DLI] and depressor anguli oris [DAO]). The table on the right-hand side of Fig. 1c shows the treatment planned for gummy smile, described using MD Codes and MD DYNA Codes. (This patient received treatment in additional facial areas; however, those codes are not included here.) These treatment steps address gummy smile by reducing the excursion of the upper lip levators to lower the smile line and elongate the upper lip, balancing the activity of the antagonistic depressors (DAO and DLI), and increasing the resistance of the orbicularis oris (OO) to prevent upper lip inversion.

The first injection was at the anteromedial cheek (Ck3); Juvéderm Voluma^®^ XC (Allergan plc, Dublin, Ireland; 1.0 mL/side) was placed above the LAN, LLS, and Zmi using an injection angle < 30°, to reduce their contraction. Voluma injected beneath the DSN, at the bone level of the premaxilla area at the nasolabial angle (N1; 0.2 mL/side), provided a mechanical block of the muscle’s contraction to reduce downturn of the tip of the nose and to reduce the lifting of the central part of the lip. Juvéderm Volift XC (Allergan plc, Dublin, Ireland) was injected above the LAN and LLS at the upper and central nasolabial fold (NLF; NL1 [0.5 mL/side] and NL2 [0.3 mL/side]), respectively, in the subcutaneous layer, and at the cutaneous part of the upper lip in the subcutaneous layer (Lp^8^; 0.25 mL/side), as well as in the subcutaneous layer above the ZMi at the lower NLF (NL3; 0.2 mL/side) to further reduce levator activity. Volift was injected under OO at the upper lip (Lp^1^; 0.25 mL/side) to support the muscle and increase its resistance against the pull of the upper lip levators. Voluma was injected above depressors at the labiomental angle (C1 [0.7 mL/side]; DLI), lower prejowl (Jw4 [0.5 mL/side]; DAO and DLI), and lower anterior chin (Jw5 [0.5 mL/side]; DLI); each of these was injected at the subcutaneous level to stretch muscle fibers in order to reduce contraction and rebalance activity with the upper lip levators. After treatment, the movement of the upper lip levators and lower lip depressor is reduced during smile, and consequently, the retraction of the upper lip and lower lip is decreased, as shown immediately after HA filler injection (Fig. 1c, center left image) and a day later (Fig. 1c, bottom left image).

The results from this case illustrate how concepts outlined in the 2018 “Myomodulation with Injectable Fillers” paper are now being put into practice using the MD Codes and MD DYNA Codes, which were developed from those concepts, to guide HA filler injection placement for mechanical modulation of muscle action. The ideas presented in the original paper are now supported in clinical practice by the use of techniques that are based on an understanding of the possible effects of HA filler injection on facial mimetic muscles and the improvements in facial appearance that can result. Going forward, the concept of mechanical myomodulation and the techniques it has inspired will be tested in formal studies to inform clinicians who might consider adopting them in their own facial aesthetics practice.

## References

[1] de Maio M (2018). Myomodulation with injectable fillers: an innovative approach to addressing facial muscle movement. Aesthetic Plast Surg.

[2] Maas C, Kane MA, Bucay VW, Allen S, Applebaum DJ, Baumann L, Cox SE, Few JW, Joseph JH, Lorenc ZP, Moradi A, Nestor MS, Schlessinger J, Wortzman M, Lawrence I, Lin X, Nelson D (2012). Current aesthetic use of abobotulinumtoxinA in clinical practice: an evidence-based consensus review. Aesthet Surg J.

[3] Carruthers J, Carruthers A, Tezel A, Kraemer J, Craik L (2010). Volumizing with a 20-mg/mL smooth, highly cohesive, viscous hyaluronic acid filler and its role in facial rejuvenation therapy. Dermatol Surg.

[4] Kane MAC (2018). Commentary on myomodulation with injectable fillers: an innovative approach to addressing facial muscle movement. Aesthetic Plast Surg.

